# Extra Limites

**Published:** 1821-06-01

**Authors:** 


The following letter was lately received, bearing the post mark of a large town in the country; and as it contains observations which we deem to be deserving of attention, we shall give it place by way of opening the eyes of ARGUS, for the first time, to the wonderful scene of human life around him.

### XVII.

### EXTRA LIMITES.

### ARGUS, No. I.

---

Centum luminibus cinctum caput ARGUS habebat,
Inque suis vicibus capiebant bina quietem,
Cætera servabant.

---

### *To the Editors of the Medico-Chirurgical Review.*

GENTLEMEN,

At page 787 of your fourth number, you have introduced an extract from an oration pronounced by Professor Richerand of Paris, in which he draws a humiliating picture of the Profession in his own country, "in consequence of the medical market being overstocked." In the succeeding page, Gentlemen, you seem to think that M. Richerand's observations will not apply to Great Britain; but probably were you to witness the measures pursued by competitors for medical celebrity in the *country*, you would alter your opinions. In the metropolis, indeed, the *odium medicum* is less observable, because the interests of individuals do not so immediately clash, and because the great mass of talent diffused through such a boundless capital, preserves a degree of equilibrium that drowns, in a great measure, those personal conflicts that disgrace medical society on more circumscribed theatres of action. In the metropolis too, a physician or surgeon seldom attains eminence without securing the esteem of his *professional* brethren by upright conduct as well as scientific acquirements; and hence he *there* finds it his interest (sooner or later) as well as duty, to act in conformity with the dictates of honour and liberality. But in the country it is very different, and too many medical practitioners make it their sole line of policy to ingratiate themselves with the public and their patients at the expense of their cotemporaries in the same profession. When a gentleman starts upon this plan, and has the means of enlisting a few non-professional gossips in his service, the scenes that ensue are really disgusting to a philosophic observer, and derogatory from the dignity which *ought* to attach to human nature.

If the theatre of this farce happen to be a place of fashionable resort, the first engine of medical manœuvring is a dashing equipage —not the fair and natural result of a gradual and progressively increasing *practice*, but the *coup-de-main*, to storm the citadel of public opinion, or the *royal road* to reputation, without the toilsome process of advancing step by step, in patient investigation, attentive observation, and laborious discharge of painful, and often unrequited duties. This piece of policy, however, would be pardonable, or rather *pitiable*—for the bubble sooner or later bursts; but the same actuating principle prompts to other and less excusable measures. The feelings and the credulity of the afflicted are tampered with, imposed upon, and turned into engines subversive of fairly earned reputation in others, or subservient to the establishment of fungous reputation for themselves. It is apparently in vain that the medical community of a place see and feel this disingenuous and unprincipled mode of conduct in some of their brethren. Their voice is disregarded by the actor, or artfully represented as the effusion of envious or jealous feeling towards rising talent and merit. What then is to be done? Are we to silently look on and see ourselves undermined and ruined; or must we adopt the same weapons in our own defence, and thus degrade a noble and venerable science, in the eyes of the world? This, Gentlemen, is the question I would ask; and this is my motive for appealing to the general tribunal of the Profession.

I am, with much respect,

Your constant Reader,

*A Country Physician.*

☞ We apprehend the evil complained of exists to a great extent, but we would recommend a stern and inflexible perseverance in the path of *honest assiduity*, as the surest road, in the long run, to honorable distinction. EDITORS.

### ARGUS, No. 2.

### OBSTINATE PERVIGILIUM.

A medical gentleman, between forty and fifty years of age, residing in the West Indies, has been twice affected with states of obstinate insomnolency, the first lasting from six weeks to two months, the last attack continuing nearly three months, without a wink of sleep. These sleepless fits come on without any preceding indisposition, of any kind, the gentleman being in perfect health. For the first week or two he feels little inconvenience; goes out to parties, and spends the nights in amusements; but after this period, a train of nervous symptoms succeeds, attended with excessive irritability, and derangements in the vascular system and secreting organs. Every means which the medical men of the island could suggest proved entirely useless, and the pervigilium went off as suddenly as it came on, without any manifest cause, and leaving the gentleman in his usual state of health. The second attack lasted so long that himself and friends became alarmed for the consequences, and after being nearly three months without a moment of sleep, he embarked in the packet for England to obtain medical advice. The packet put to sea in the evening, and the gentleman fell fast asleep, and continued so twelve hours, since which (six or eight months) he has his usual rest, and is in good health. This gentleman has been seen by several physicians in London, none of whom could discover any symptom of organic or other disease to account for the two attacks abovementioned. He is desirous of obtaining opinions on his case, and of learning whether any gentleman has met with any thing similar in his practice.

---

### ARGUS, No. 3.

### *The Surreptitious Knight:* or, *Sir Charlatan Glanderman.*

Sir, *Blackheath,* 15*th May,* 1821.

As Editor of the Medico-Chirurgical Review, I conceive it to be your duty, as well as inclination, to expose the various systems of quackery resorted to in the Metropolis, from the well-known Dr. Eady, of Privy Wall notoriety, up to the equally celebrated prognosticator of a Shiloh, and his honorable and literary friend Sir ——————*

I shall not therefore waste either my time or paper in apologizing for endeavouring to give you some idea of the claims, which the last-mentioned worthy has to the *distinction* he enjoys. To disclose to you his *particular merits,* and the manner in which I was so unfortunate as to become acquainted with them, is the object of my present letter. In the first place it will be necessary to inform you, that I am not one of the profession, which will be some excuse for my ignorance; in the next,

---

* *We have suppressed the name of the Gentleman (if such a disgrace to the Profession deserves that appellation) who forms the subject of Argus's glance in this paper.*

that an inclination for literature, which prompts me to read every thing which comes in my way, was the means of introducing me to the acquaintance of Sir ———. Turning over the pages of the ——— ——— for ——— last, my attention was arrested by a short review of a work professing to be written by Mr. ———, on ——— ———, &c. Being of a scrofulous habit, I was induced to peruse this review, with its accompanying parts of the work, in which cases were cited to have been cured of a most extraordinary and desperate kind, by his peculiar treatment. Little suspecting that the conductors of a respectable periodical publication would let their pages out to hire to any advertizing Charlatan, I was silly and credulous enough to determine upon waiting on the *Author*, for the purpose of submitting my case to his attention. I accordingly waited on him, and told him whence my visit originated; and that I found what he had observed in his work with regard to the tendency of mercury in my disorder, was perfectly correct.—" Yes," he replied, " IT IRRITATE THE SYSTEM :" and immediately he entered into a long tirade against mercury and its general use by the profession as an universal panacea. His intention was evidently to astonish me, and so he did—for judge of my surprise at finding our author setting all the rules of syntax at defiance; in short, Sir, without being a critic, I perceived that amidst his numerous cases, that of the nominative came least under his attention. I was the more astonished at seeing the table covered with classic authors, and the room surrounded with models of arms, legs, feet, &c. all tending to impress the poor patient with the idea of a cognoscenti. Not to intrude too much on your time, he gave me some medicine, desired me to call again in a fortnight, and assured me that I had only to persevere in taking his medicines to effect a permanent cure. At the same time he put a card into my hand, which I have lost, but which I believe was as follows:—" Mr. ———, Surgeon, Accoucheur, ———House, ——— Square. C———r, S———a, and Diseases of the Breast, particularly attended to." I called the same morning on a friend who resides in town, to whom I related what had passed, adding my conviction that he must have employed some Grub Street hack to write the pamphlet for him. My friend observed, " that he never heard any thing respecting Mr. ———, but that he was sufficiently convinced of his celebrity from his card, as no regularly bred practitioner would resort to such means for attracting public notice; I would recommend you to throw away his medicine, and to congratulate yourself on getting so cheaply out of his hands." Unfortunately I neglected my friend's recommendation, and the result will be best explained by the subjoined correspondence, in which I have taken particular pains to preserve the original orthography of Sir ———.

*From Mr. ———, accompanied by a bill of great length.*

" DEAR SIR,

I have *send* you some more *altrertive* powders, and hope they are of service—Next week I may probably encrease the power of what you are taken—I send you the account as it stands.

Your's truly, ———."

*To Mr. ———, in reply to the above.*

" SIR,

I cannot find words to express the astonishment which I felt yesterday morning at the receipt of your bill, amounting to seven pounds eight shillings and six pence. I applied to you for advice, as I informed you on my first visit, in consequence of having seen in a respectable periodical publication a favourable review of a work on ——— diseases, *purporting to be written by you.* I was more particularly induced to consult you from seeing the respectable name of Mr. Astley Cooper adduced, as having witnessed a cure which you performed, and which his well-known skill had failed to effect. As a total stranger to you I thought proper, on my first visit, to ask how much I was indebted to you; you replied "you may leave a pound," which I did; I left another on my next visit, and my purse underwent the same pull on the fourth of October, when I last *had the pleasure of seeing you,* and in so doing I considered that I had settled *all claims up to that period.* Could I have supposed for a moment that I was to pay at the rate of sixteen shillings and sixpence per bottle, from that moment I should have discontinued my visits, as neither my purse nor my inclination would have suffered me to take cordials so expensive. Five shillings I considered would be the utmost you could possibly charge. Experience, however, makes even wise men wiser; and this expensive lesson will, in future, prevent me from setting limits to that which is illimitable—the exorbitant conscience of an apothecary.* Why, Sir, you really surpass the utmost skill of the antients, who sought to transmute baser metals into gold; your flights are far more daringly elevated, and your success is hand in hand with your ambition, when you succeed in extracting that precious ore from a trifling discolouration of the simple element.

*It was your business to have apprized me of the inadequacy of my reward in the first instance, when I should immediately have supplied the deficiency,* and have discontinued my visits, and not have been mortified and insulted by a preposterous bill sent in without any preparation on my part for its receipt. My income does not amount to more than six shillings per day, and I appeal to yourself whether in common prudence I could have been so egregiously stupid as to swallow more than half that amount in about a quarter of a pint of medicine. As you took upon yourself to send in the bill, you might, in common delicacy, have kept back the last bottle, more particularly after a week's delay. This is the first time in my life that I ever felt the least reluctance in discharging a bill; on the contrary, I have ever derived more pleasure in defraying than in contracting an account; and had the amount of yours been at all reasonable, I should have paid it without hesitation. You, however, well know that it is not reasonable, and to my sorrow I am sufficiently acquainted with the routine of medical practice, to be aware that three shillings per dozen for powders is a very ample remuneration, *when there is no attendance.* In your bill you charge me for powders four pounds and upwards, which I will not pay but "on compulsion," as I deem it more honorable to resist, than to submit to, an unjust demand. Rather, however, than be involved in legal squabbles, I will immediately transmit you three pounds in addition to what I have already paid you, provided that you send me a receipt in full of your demand. Awaiting your reply,

I am, &c.

*Letter in reply to the above, in which Sir Charlatan endeavours to be facetious.*

"Sir, "7th Nov. 1820.

Had you thought correctly, (and I am not answerable for your thinking wrong,) or even shewn my account to any respectable medical

---

* *The victim of the unprincipled Charlatan exposed in this letter should not identify the impositions of a quack with the proper charges of an apothecary.* Ed.

man, he would have informed you my charges are as common as they are just. You *can not* therefore have added "wisdom to the wise" as you are perfectly ignorant of what you talk of. Of your *limitted* income I have *nothing to do it,* and I assure you I have been too long established and I hope too respectably situated than to wish to impose on any one and I can not have wounded your delicacy as the tenour of your letter satisfies me on that head. I am, Sir, ———."

This note is so much better expressed than either of his former ones, that I feel assured that it was dictated for him, and that his time was too much occupied to enable him to *copy* it correctly. Be this as it may, being informed that as medical charges were, for the most part, arbitrary, the law would compel me to pay, not only the bill, but all other expenses; under these circumstances my poverty, rather than my will, prompted me to send him the note which follows.

"To Mr. ———.

"Sir,

I have supplied the bearer with the amount of your bill, and without entering into any further controversy, *I leave it to your sense of justice*, either to take the whole amount, or to make that deduction which a candid consideration must necessarily dictate. I reiterate all my *former* assertions, and am, Sir, &c."

It will be readily imagined that my appeal was to a nonentity. Thus ended a correspondence, which discovers that the lottery contractors do not engross the whole of the puffing system, and that education is not essential to knighthood. To the many ornaments of the profession, who have hitherto contributed to render the honour respectable, I fear the name of Sir Charlatan will be considered rather as an addition than an improvement to the order. You are of course at liberty to make what use you please of this communication, and by intimating a wish to that effect, I will readily supply you with the Knight's original letters. The galled jade will naturally wince, but I assure you I am induced to address you, more from a desire of pushing presumptious Ignorance from her throne of brazen impudence, than from any vindictive feeling towards the contemptible individual who equally disgraces knighthood and the Faculty. I am, Sir, with great respect,

M———."

☞ We have thought it our bounden duty to give insertion to the above letter, in order that our readers may shew it to such of their neighbours as may have been equally duped by the above or any other of the numerous quacks which infest this metropolis. At the same time we cannot commiserate men of such sense and education as M. evidently is, when they run headlong into the jaws of Charlatanism, and thus place that confidence in ignorance and impudence which they refuse to talents and candour. Editors.

